# Are parenting programmes effective at scale? Associations with violence against adolescent girls, parenting and mental health in real-world delivery across eight African countries: a meta-analysis of pre-post surveys

**DOI:** 10.1136/bmjgh-2025-020422

**Published:** 2026-05-05

**Authors:** Lucie Cluver, Catherine L Ward, Francesca Little, Inge Vallance, Genevieve Haupt Ronnie, Yulia Shenderovich, Hlengiwe Gwebu, Kufre Joseph Okop, Frances Gardner, Lindokuhle L Ngcobo, Mark Tomlinson, Daniel Oliver, Zuyi Fang, Natalie Davidson, Roselinde Janowski, Heiletjé Van Zyl, Anna Booij, Nyasha Manjengenja, Sibongile Tsoanyane, Muhubiri Kabuyaya, Mukondi Nethavhakone, Tendai Mutembedza, Alison Koler, Amon Exavery, Anne Schley, Charles Bibuya, Daisy Kisyombe, Esther Nydetabura, Farai Charasika, Gideon Mavise, Henry Mbuyi, Jack Ngangula, Jeldau Rieff, Joyce Wamoyi, Lisa Jamu, Nomsa Monare, Richard Savo, Samuel Bojo, Styn Jamu, Thomas Kipingili, Vengai MacGerald Mujuru, Jamie Lachman

**Affiliations:** 1 Department of Social Policy and Intervention, University of Oxford, Oxford, UK; 2 Department of Psychiatry and Mental Health, University of Cape Town, Western Cape, South Africa; 3 Centre for Social Science Research, University of Cape Town, Rondebosch, Western Cape, South Africa; 4 Department of Psychology, University of Cape Town, Western Cape, South Africa; 5 Department of Statistical Sciences, University of Cape Town, Western Cape, South Africa; 6 Nuffield Department of Primary Health Sciences, University of Oxford, Oxford, UK; 7 Wolfson Centre for Young People’s Mental Health, Cardiff University, Cardiff, Wales, UK; 8 Centre for the Development and Evaluation of Complex Interventions for Public Health Improvement (DECIPHer), School of Social Sciences, Cardiff University, Cardiff, UK; 9 Department of Nursing and Public Health, University of Fort Hare, East London, South Africa; 10 Department of Prevention and Evaluation, Leibniz Institute for Prevention Research and Epidemiology - BIPS, Bremen, Germany; 11 Department of Medicine, University of Cape Town, Cape Town, Western Cape, South Africa; 12 Clowns Without Borders South Africa, Pietermaritzburg, South Africa; 13 Institute for Life Course Health Research, Department of Global Health, Stellenbosch University, Stellenbosch, Western Cape, South Africa; 14 School of Nursing and Midwifery, Queen’s University Belfast, Belfast, UK; 15 Catholic Relief Services USA, Baltimore, Maryland, USA; 16 Room to Read, San Francisco, California, USA; 17 Institute of Population Research, Peking University, Beijing, Beijing, China; 18 Center for Neurogenomics and Cognitive Research, Department of Complex Trait Genetics, Vrije Universiteit Amsterdam, Amsterdam, The Netherlands; 19 Clowns Without Borders South Africa, Pietermartzburg, South Africa; 20 University of Lubumbashi, Lubumbashi, Katanga, The Democratic Republic of the Congo; 21 Department of Psychology, University of Cape Town, Rondebosch, Western Cape, South Africa; 22 Pact Tanzania, Dar es Salaam, United Republic of Tanzania; 23 mothers2mothers, Mbombela, Western Cape, South Africa; 24 Catholic Relief Services, Kinshasa, The Democratic Republic of the Congo; 25 Strategic Information, Pact Eswatini, Mbabane, Hhohho, Eswatini; 26 Catholic Relief Services Zimbabwe, Harare, Zimbabwe; 27 FHI 360, Harare, Zimbabwe; 28 Pact Zambia, Lusaka, Zambia; 29 Stepping Stones International, Gaborone, Botswana; 30 Trauma Aid Netherlands, Amsterdam, The Netherlands; 31 National Institute for Medical Research Mwanza Research Centre, Mwanza, United Republic of Tanzania; 32 Agency for Research and Development Initiative, Juba, Central Equatoria, South Sudan

**Keywords:** africa, global health, mental health & psychiatry, violence, child health

## Abstract

**Introduction:**

Evidence-based parenting programmes are widely used to prevent violence against children and improve parenting and mental health. Despite hundreds of randomised trials, little is known about their outcomes when delivered at scale within routine delivery. This study assesses the WHO-endorsed and UNICEF-endorsed Parenting for Lifelong Health programme for caregivers and adolescents, delivered through non-governmental organisation and government in Botswana, the Democratic Republic of the Congo, Eswatini, South Africa, South Sudan, Tanzania, Zambia and Zimbabwe, with support from the President’s Emergency Plan for AIDS Relief (PEPFAR), the United States Agency for International Development (USAID) and the European Union.

**Methods:**

Pre-post surveys for caregivers and adolescents were integrated into service data collection between 2016 and 2022. Abbreviated standardised measures of physical abuse, emotional abuse, approval of corporal punishment, positive involved parenting, monitoring/supervision, caregiver depressive symptoms, parenting stress and adolescent depressive symptoms and externalising behaviour were used. Individual country scores were analysed separately for caregivers and adolescents using generalised linear mixed-effects models, and cross-country data were combined using a random-effects meta-analytic model.

**Results:**

123 050 participants were included (93% retention, 57 908 adolescents (96% female), 56 423 caregivers at follow-up). In all-country meta-analyses, estimates showed reduced physical abuse (−65%; 95% CI 51% to 74%), emotional abuse (−59%; 95% CI 48% to 68%) and approval of corporal punishment (−55%; 95% CI 48% to 60%). Positive involved parenting increased (+52%; 95% CI 24% to 87%) and poor supervision/monitoring decreased (−48%; 95% CI 34% to 58%). Caregiver depressive symptoms (−25%; 95% CI 8% to 48%), parenting stress (−46%; 95% CI 41% to 52%), adolescent depressive symptoms (−22%; 95% CI 1% to 38%) and adolescent externalising behaviour problems (−43%; 95% CI 29% to 54%) all declined. There was heterogeneity in pre-intervention scores and extent of change between humanitarian and development settings, and between different target groups, but strong consistency across caregiver and adolescent reports.

**Conclusion:**

In eight African countries, including humanitarian and pandemic-affected contexts, an evidence-based parenting programme showed consistent associations with reduced violence against adolescent girls and improved parenting and mental health.

**WHAT IS ALREADY KNOWN ON THIS TOPIC**

- There has been increased uptake of evidence-based parenting programmes by governments, non-governmental organisations (NGOs) and international agencies to prevent violence against children; however, there is scant research demonstrating that parenting interventions remain effective when delivered at large scale, through real-world systems in Global South, and in humanitarian contexts.
- The effectiveness of parenting interventions is often compromised when they transition from well-managed randomised trials to large-scale delivery.

**WHAT THIS STUDY ADDS**

- This study shows that an evidence-based parenting programme for caregivers and their adolescents can retain effectiveness when delivered at scale within routine NGO and government delivery in eight African countries, including in humanitarian and pandemic-affected contexts.
- This study highlights the intervention’s substantial positive impact on violence prevention, parenting and mental health, with remarkable consistency in reported positive effects across countries and between caregiver and adolescent reports.

**HOW THIS STUDY MIGHT AFFECT RESEARCH, PRACTICE OR POLICY**

- This study adds further support to the movement for governments to provide evidence-based parenting programmes as a means to tackle violence against children across contexts, including humanitarian settings.
- It also highlights the value in implementing partners embedding outcome measures within routine monitoring and evaluation processes so that effectiveness can be monitored as interventions are delivered widely.

## Introduction

 Evidence-based parenting programmes are now recognised as a primary approach for prevention of violence against children.^1^ The WHO guideline reviews on parenting interventions identified over 400 randomised trials,^2^ with >150 in the Global South,^3^ although reported limited evidence with families of adolescents, and in humanitarian contexts.^4^

This evidence has contributed to increasing uptake of parenting programmes by national governments, non-governmental organisations (NGOs) and international agencies, including UNICEF, WHO and the UN Office on Drugs and Crime, and through US government-funded foreign assistance.^5^ In 2024, over 50 governments made commitments to deliver parent and caregiver support at the First Global Ministerial Conference on Ending Violence Against Children.^6^

Evidence-based parenting programmes use structured sessions, delivered primarily either to groups of caregivers or individually through home visits, which aim to build caregiver-child relationships, reduce violent discipline and improve child behaviour. Across different curricula, they share non-didactic social learning theory approaches, developing parenting skills such as praise, one-on-one time, family rules and alternatives to violent discipline. Throughout this field, ‘parenting’ and ‘parents’ are understood as referring to any primary caregiver of a child or adolescent.

Despite substantial evidence from well-controlled experiments, very little is known about whether parenting programmes remain effective when delivered at scale through real-world systems in Global South and humanitarian contexts.^7^ This is particularly important, because it is common for interventions to lose effectiveness when transitioned from well-managed randomised trials to large-scale delivery.^8^ A recent review^9^ found a small number of ongoing studies evaluating scaled-up delivery, but only among early childhood programmes.^10 11^

Real-world delivery inevitably brings changes and challenges to implementation fidelity. These include budget constraints, delivery alongside other services and differing skills and training of staff.^12^ In crisis contexts, such as conflict, climate hazards and epidemics, additional challenges include service interruptions and lockdowns.^13^ It is therefore essential to understand whether parenting programmes can sustain effects when delivered in such contexts.

Africa is a region of key importance because of its rapidly rising adolescent population, estimated to reach half a billion by 2050.^14^ In particular, adolescent girls in Africa face high rates of HIV infection,^15^ associated with elevated violence victimisation^16^ and mental health distress.^17^ Non-violent and positive parenting are associated with reduced risks,^18^ but families face extreme stressors including the world’s highest rates of poverty,^19^ armed conflict and natural hazards.^20^

Parenting for Lifelong Health (PLH) is a suite of freely available parenting programmes developed by researchers, with WHO and UNICEF,^21^,^23^ tested in 15 randomised trials across Africa, Asia and Eastern Europe.^24^,^28^ PLH for adolescents was first tested in a cluster randomised trial in South Africa between 2012 and 2015,^24^ and includes core parenting skills, mindfulness-based mental health support, family budgeting, planning for adolescent safety and family communication around HIV risks. Delivered in 40 countries, PLH programmes are now some of the most widely scaled parenting programmes globally.

This study aimed to better understand scale-up of PLH programmes in real-world conditions.^22^ Focusing on adolescents aged 10–17 years and their caregivers, who currently make up the largest proportion of programme recipients in the African region, it assesses whether caregivers and adolescents report changes in violence, parenting and mental health after completing a parenting programme and conducts meta-analyses across countries to investigate associations across differing contexts.

The study also aims to build evaluation capacity among NGOs and governments that implement parenting programmes, who increasingly need to assess effects within standard programme delivery but lack methodological support and experience. In each country, implementing organisations received training on evaluation procedures and were supported to clean and store their data, analyse and write up findings, including combining quantitative findings with qualitative insights.^29 30^

Research questions were: (1) Across countries, service providers and target populations, do caregivers and adolescents report changes in violence, parenting and mental health after programme receipt? (2) To what extent do caregiver and adolescent reports concur? (3) Are there overall impacts across a highly heterogeneous region?

## Method

### Patient and public involvement

Patients and the public were not involved in the design, conduct, reporting or dissemination of this research. Local implementing partners (NGOs and government agencies) did, however, review survey tools for cultural and linguistic relevance and were trained and supported to collect and analyse data.

### Context and description

**Figure 1:**
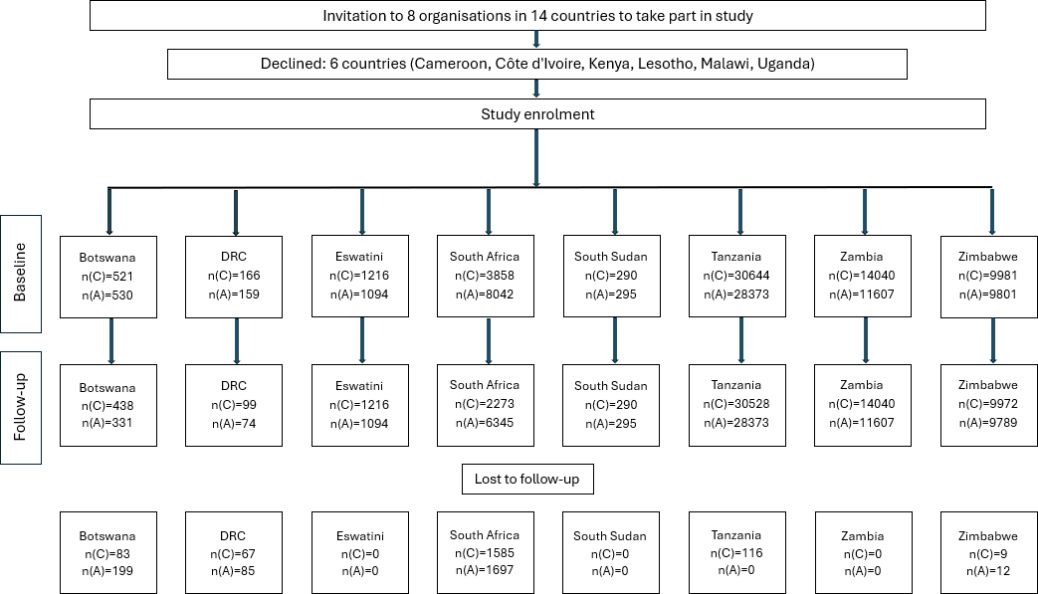
Flow chart of study participation. DRC = the Democratic Republic of the Congo; n(C) = Number of Caregivers; n(A) = Number of Adolescents.

From 2016 to 2019, the study was discussed with implementing partners in countries that were planning delivery of PLH for adolescents. Participation was entirely voluntary, and agencies received a small amount of funding and training to support data collection activities from 2016 to 2022. Eight countries took part: Botswana, the Democratic Republic of the Congo (DRC), Eswatini, South Africa, South Sudan, Tanzania, Zambia and Zimbabwe. Implementers in six other countries: Cameroon, Côte d’Ivoire, Kenya, Lesotho, Malawi and Uganda lacked staff capacity to participate (figure 1). We note that as the programmes are freely available (on the WHO and PLH websites), it is likely that additional implementation also took place of which the research team was unaware.

The programmes were delivered in real-world conditions, which included armed conflict (DRC and South Sudan), civil violence (in South Africa and Eswatini) through the pandemic of COVID-19 (in Botswana, Eswatini, South Africa and Tanzania) and the HIV/AIDS epidemic and through natural hazards including drought and flooding. For example, in Botswana, South Africa and Zimbabwe, implementation was transferred to phone and WhatsApp delivery during COVID-19 lockdowns, but remote programme delivery was hampered by lack of internet connectivity and access to data.

### Sample and data

Each country selected target populations based on their programme remit and budget, and their target population, for example, South Sudan aimed to reach adolescents orphaned by HIV/AIDS and conflict, while Tanzania focused on adolescent girls in high HIV-prevalence areas. In each country, partner organisations were trained in survey implementation, minimisation of bias and data collection. Pre-post surveys were integrated into routine service data collection. Matched caregiver and adolescent dyads were enrolled (except in South Africa, where caregiver and adolescent recruitment were independent) in 14-week intervention programmes. In several contexts, the PLH programme was embedded in a larger suite of interventions (such as PEPFAR DREAMS (Determined, Resilient, Empowered, AIDS-free, Mentored, and Safe) and Orphaned and Vulnerable Children (OVC) programmes, a European Union programme), but implementation, selection of participants and assessment were similar across countries, allowing combination in a meta-analysis. All available pre-post records were included after data cleaning, and sample sizes were determined pragmatically, based on programme coverage and operational capacity rather than formal power calculations (figure 1).

The study protocol was published,^31^ and ethical approval was granted in all countries involved. Partner organisations received capacity training in ethical research processes including informed consent. Each country retained and stored its own primary data, sharing only an anonymised version for meta-analysis through data sharing agreements. The analyses involved (1) individual-level models fitted within each country and (2) country-level coefficients combined in a random-effects meta-analysis.

### Measures

Self-report questionnaires were completed by caregivers and adolescents. All implementers highlighted the need for brevity, and so we conducted factor analysis on data from previous trials^24^ to provide reduced versions of standardised measures and brief socio-demographic questions. All scales were translated and back-translated into local languages and answered separately by caregivers and by adolescents (except where noted below). As each organisation led their own research, there was minor variation in items across countries, as well as within countries where different implementing organisations were delivering the programme independently. Consequently, we used items within each scale that were measured consistently across seven or more countries (31 items) and noted any discrepancies below.

#### Violence against children

*Physical abuse* (eight countries) was measured using two items from the International Society for the Prevention of Child Abuse and Neglect Screening Tool for Trials (ICAST-Trial) physical abuse subscale,^32^ based on frequency in the past 4 weeks. Caregiver items were “How often did you discipline your child by spanking, slapping or hitting them with your hand?” and “How often did you discipline your child with an object like a stick or a belt?” Adolescents responded to the same items, for example, “How often did your caregiver…?” *Emotional abuse* (eight countries) was measured using two items from the ICAST-Trial: “How often did you shout, yell or scream at your child?” and “How often did you say angry things to your child that upset him/her?” Measures were consistent across all countries, except Zimbabwe where only the second item was used. *Approval of corporal punishment* (eight countries) was measured using one Likert-scale item from UNICEF’s Multiple Indicator Cluster Survey^33^: “In order to bring up, raise, or educate a child properly, they need to be physically punished”.

#### Parenting

*Positive involved parenting* (eight countries) was measured using three items from the Alabama Parenting Questionnaire subscale,^34^ based on frequency in the past 4 weeks: “You have a friendly talk with your child”; “You get involved in activities that your child likes”; “You talk to your child about his/her friends”. Measures were consistent across all countries. *Poor supervision/monitoring* (eight countries) was measured using three items from the Alabama Parenting Questionnaire subscale: “Your child stays out in the evening past the time when he/she is supposed to be home”; “Your child goes out without a set time to be home”; “Your child goes out after dark without an adult with him/her”. Measures were consistent across all countries, except for Eswatini, where only the second item was used.

#### Mental health

*Caregiver depressive symptoms* (eight countries) were measured using three caregiver-reported items from the Center for Epidemiological Studies Depression (CES-D) Scale^35^: “How often in the past week have you felt depressed?”; “How often in the past week have you felt that everything you did was an effort?” and “How often in the past week have you felt lonely?” Measures were consistent across all countries. *Parenting stress* (four countries) was measured using two caregiver-reported items from the Parental Stress Scale,^36^ based on frequency in the past 4 weeks: “Caring for your children sometimes takes more time and energy than you have to give” and “Your children are a major source of stress in your life”. Measures were consistent across all countries. *Adolescent depressive symptoms* (four countries) were measured using three items from the CES-D. Measures were consistent across all countries, except for Eswatini where the third item was removed. *Adolescent externalising behaviour* (eight countries) was measured using a single caregiver-reported item from the Strengths and Difficulties Questionnaire^37^: “Your child often has temper tantrums or hot tempers”.

### Statistical analysis

Analyses combined pragmatic real-world data with meta-analysis methods. First, data were cleaned to delete duplicates, inconsistent information, implausible ranges and non-varying responses. In some countries, data with non-unique identification numbers were deleted. In Eswatini and South Sudan, some individual prescores and postscores could not be matched and were excluded. Data for all caregivers and adolescents were included, regardless of whether caregiver and adolescent data could be matched under the assumption that all enrolled caregivers and adolescents were offered and received the intervention and that their responses would be independent of one another. This avoided bias that could have arisen by excluding data from countries with less stable political and socio-economic environments. Data were also included regardless of whether pre-intervention and post-intervention assessments were available for all participants, and missing data were dealt with through maximum likelihood estimation under the assumption of missing at random.

Second, bar graphs were used to illustrate the item-specific Likert-scale responses pre-intervention and post-intervention. Total scores for measurement scales were calculated based on items common to all countries (where a single country had missed an item, we multiplied existing items pro rata). Scores were summarised by country for caregivers and adolescents, separately, using cross-sectional means, SD, medians and IQRs and Cronbach’s α statistics pre-intervention and post-intervention.

Third, individual country scores were analysed separately for caregivers and adolescents using generalised linear mixed-effects models with subject-specific random effects to estimate the relative improvement from pre-intervention to post-intervention, taking into account within-subject repeated measurements. Statistical distributions were selected based on the Generalised Akaike Information criterion and an assessment of model fit based on residuals, and included negative binomial, generalised Poisson, Tweedie, lognormal and t-family distributions. The log link was used so that the exponentiated model coefficients provided a ratio estimate of post-intervention versus pre-intervention scores.

Fourth, models were adjusted for caregiver and adolescent gender and age, as well as whether the caregiver was a biological parent of the adolescent. Other caregiver and adolescent socio-economic and health characteristics were summarised but not included in models since they were not uniformly measured across countries.

Fifth, model results from the different countries were combined using a random-effects meta-analytic model.^38^ Inverse weighting was used to incorporate precision of study-specific estimates. Between-study heterogeneity was estimated using the I^2^ statistic by Higgins and Thompson. Participant type (caregiver vs adolescent) was included as a subgroup in the random-effects meta-analytic model and subgroups were compared using Cochran’s Q-statistic. The country, participant-specific and combined estimates from meta-analytic analyses are illustrated as forest plots. Additionally, we calculated percentage improvement in scores from pre-intervention to post-intervention. Meta-analysis results are reported using forest plots showing the relative improvement of scores from pre-intervention to post-intervention for the different country-participant strata and the combined effect. Prediction intervals that would take this effect outside the range of studies used are also shown but should be interpreted with care given the non-randomised nature of the study. Meta-analyses of pre-intervention scores are reported in the online supplemental material and can be used to anchor the relative effect estimates.

Since there was no random selection of participants, and since sample sizes were large, the focus was not on statistical inference but rather on the estimation of the size of changes following implementation of the intervention. Observed p values are reported but they are not categorised as ‘significant’ or ‘not significant’.

## Results

The programme was delivered across eight countries through various organisations (table 1), including local NGOs such as the Agency for Research and Development Initiative in South Sudan, mothers2mothers in South Africa and local offices of international NGOs, such as Catholic Relief Services in the DRC and Stepping Stones International in Botswana. In all countries, government partners were involved in the implementation, and in Tanzania and South Sudan, programmes were delivered through government services such as school and Ministry of Health staff. Among several funders, the most common was PEPFAR-USAID.

**Table 1:** Sample characteristics

Country	Implementer	Service programme	Dates of programme delivery	Participant	Time		Gender*		Age	Biological parent
					Pre	Post	Female	Male	Mean (SD)	
Botswana	Stepping Stones International	European Union Debswana	August 2020–April 2021	Caregiver	521	438	488 (94%)	33 (6%)	40 (11)	332 (72%)
				Adolescent	530	331	316 (60%)	214 (40%)	13 (2)	
				Total	1051	769				
DRC	Catholic Relief Services	USAID 4Children Esengo programmes	January 2016–December 2018	Caregiver	166	99	134 (81%)	32 (19%)	45 (11)	145 (87%)
				Adolescent	159	74	86 (54%)	72 (46%)	14 (2)	
				Total	325	173				
Eswatini	Pact	USAID Insika Kusasa		Caregiver	1216	1216	757 (62%)	456 (38%)	37 (19)	924 (76%)
			October 2020–September 2021	Adolescent	1094	1094	549 (50%)	545 (50%)	14 (2)	
				Total	2310	2310				
South Africa	mothers2mothers	PEPFAR Children & Adolescents are My Priority	May–July 2021	Caregiver	3858	2273	3760 (97%)	98 (3%)	40 (10)	3304 (86%)
				Adolescent	8042	6345	8023 (99.8%)	19 (0.2%)	13 (2)	
				Total	11 900	8618				
South Sudan	Agency for Research and Development	USAID 4Children	January 2017–January 2018	Caregiver	290	290	201 (70%)	88 (30%)	39 (9)	248 (86%)
				Adolescent	295	295	155 (53%)	140 (47%)	13 (3)	
				Total	585	585				
Tanzania	Pact	USAID Kizazi Kipya Project	June 2016–December 2021	Caregiver	30 644	30 528	19 471 (64%)	10 905 (36%)	44 (12)	24 731 (86%)
				Adolescent	28 373	28 373	28 373 (100%)	0 (0%)	12 (2)	
				Total	59 017	58 901				
Zambia	Pact	USAID Zambia Community HIV Prevention Project	April 2020–September 2022	Caregiver	14 040	11 607	13 112 (93%)	922 (7%)	39 (11)	8617 (61%)
				Adolescent	14 040	11 607	14 040 (100%)	0 (0%)	14 (2)	
				Total	28 080	23 214				
Zimbabwe	Catholic Relief Services	USAID Pathways Project DREAMS and OVC	October 2019–September 2022	Caregiver	9981	9972	9174 (92%)	674 (7%)	45 (13)	6289 (64%)
				Adolescent	9801	9789	8506 (87%)	1294 (13%)	13 (2)	
				Total	19 782	19 761				
Total				Caregiver	60 716	56 423	47 097 (78%)	13 208 (22%)	43 (12)	44 590 (76%)
				Adolescent	62 334	57 908	60 048 (96%)	2284 (4%)	13 (2)	
				All	123 050	114 331	107 145 (87%)	15 492 (13%)		

* Discrepancy with total numbers is due to missing data on gender.

There were 123 050 participants at baseline and 114 331 at follow-up (93% retention), from eight countries. At follow-up, the sample included 57 908 adolescents and 56 423 caregivers (table 1). The mean age of adolescents at baseline was 13 years (SD=2 years) and 96% were female, primarily due to delivery within broader programmes focusing on adolescent girls. The mean age of caregivers was 43 (SD=12 years); 78% were female; 76% were biological parents of the adolescents and the remainder were primarily grandparents. Details of data cleaning are given in online supplemental material S4.

The study sites reflected a range of socio-economic, health and education experiences, although socio-demographic variables were not collected in the DRC and only for a small subsample of caregivers in South Africa. All programmes were targeted at highly vulnerable groups within that country’s context. For example, over half of adolescents in six countries were food insecure, rising to 96% in South Sudan; and 10%–36% of households had an adult member who was hospitalised or bedridden. In Botswana, Eswatini, South Africa and Zimbabwe, around a quarter of households had at least one HIV-infected member, rising to 64% in South Sudan. Household alcohol or drug use ranged between 29% and 52%, except for Tanzania (14%), and 6%–17% of households had a family member with a severe disability.

Online supplemental figure 4a-d shows bar charts for the pre-intervention and post-intervention item responses. In most cases, improvements are observed. Online supplemental table 1a and b presents cross-sectional summary statistics, including Cronbach’s α for the different scales pre-intervention and post-intervention. In most cases, substantial decreases in mean scores are observed post-intervention compared with pre-intervention. Cronbach’s α statistics vary and are sometimes low, reflecting the fact that many of the scores were based on as few as two items.

**Figure 2:**
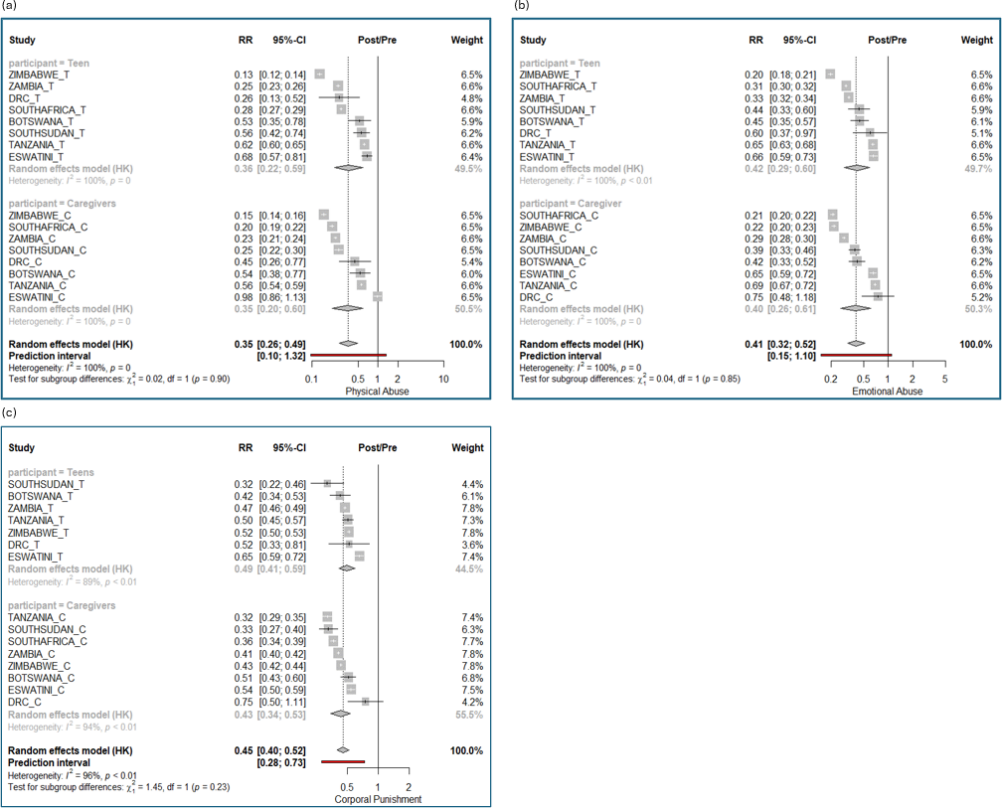
(a) Violence against children: meta-analytic estimates of change by country in scores from pre-intervention to post-intervention in physical abuse. (b) Violence against children: meta-analytic estimates of change by country in scores from pre-intervention to post-intervention in emotional abuse. (c) Violence against children: meta-analytic estimates of change by country in scores from pre-intervention to post-intervention in corporal punishment. HK = Hartung-Knapp; RR = Risk Ratio, in this analysis measuring the proportional change in the measurement score from pre- to post-intervention.

Caregiver and adolescent outcomes across countries were combined using meta-analytic methods (figures2^5^). To facilitate interpretation, we describe as an example the multicountry meta-analysis for physical abuse (figure 2a). The risk ratio columns present the ratio estimates (and 95% CI) of post-intervention over pre-intervention levels. The weight column indicates the relative weight that each country-participant type (caregiver/adolescent) stratum contributed to the combined estimate. All countries were weighted equally except for the DRC and Botswana, who contributed less to the estimate of the combined result. The heterogeneity results show substantial between-country heterogeneity; nonetheless, the country-specific estimates indicate a consistent reduction in physical violence scores across all adolescent and caregiver reports, apart from the estimate for caregiver report in Eswatini, which showed no effect. The subgroup comparison showed no difference between results for caregivers and adolescents (p=1.00). The overall combined ratio of post-intervention to pre-intervention of 0.35 (95% CI 0.26 to 0.49) translates into a 65% decrease in physical violence scores from pre-intervention to post-intervention.

**Figure 3:**
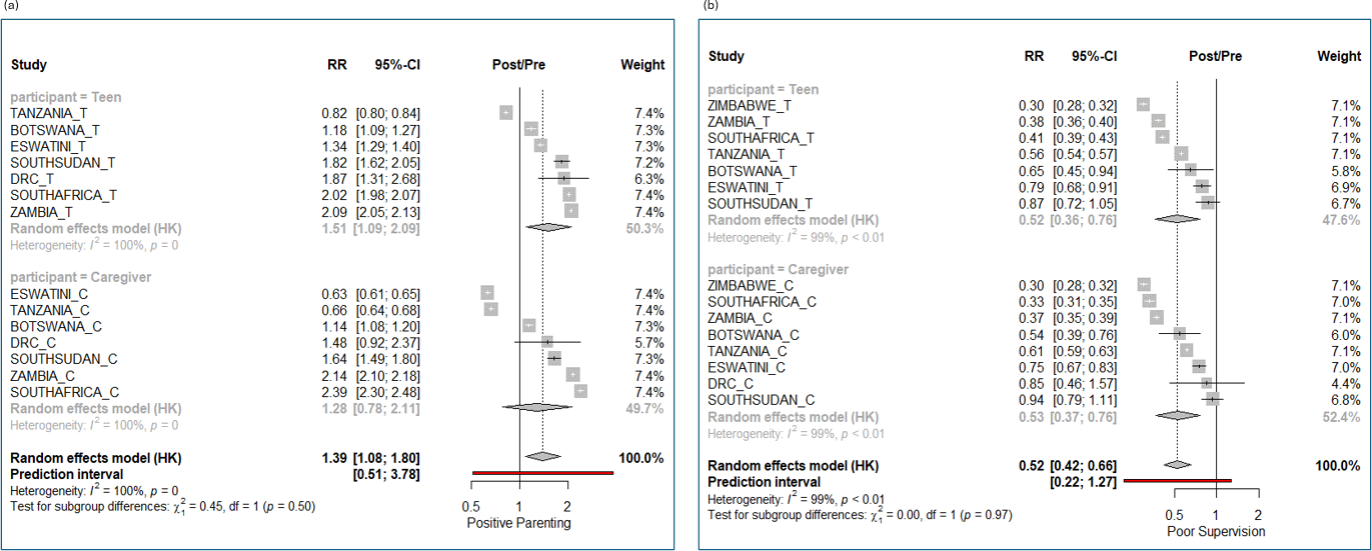
(a) Parenting: meta-analytic estimates of change by country in scores from pre-intervention to post-intervention in positive involved parenting. (b) Parenting: meta-analytic estimates of change by country in scores from pre-intervention to post-intervention in poor parental monitoring/supervision. RR = Risk Ratio, in this analysis measuring the proportional change in the measurement score from pre- to post-intervention.

**Figure 4:**
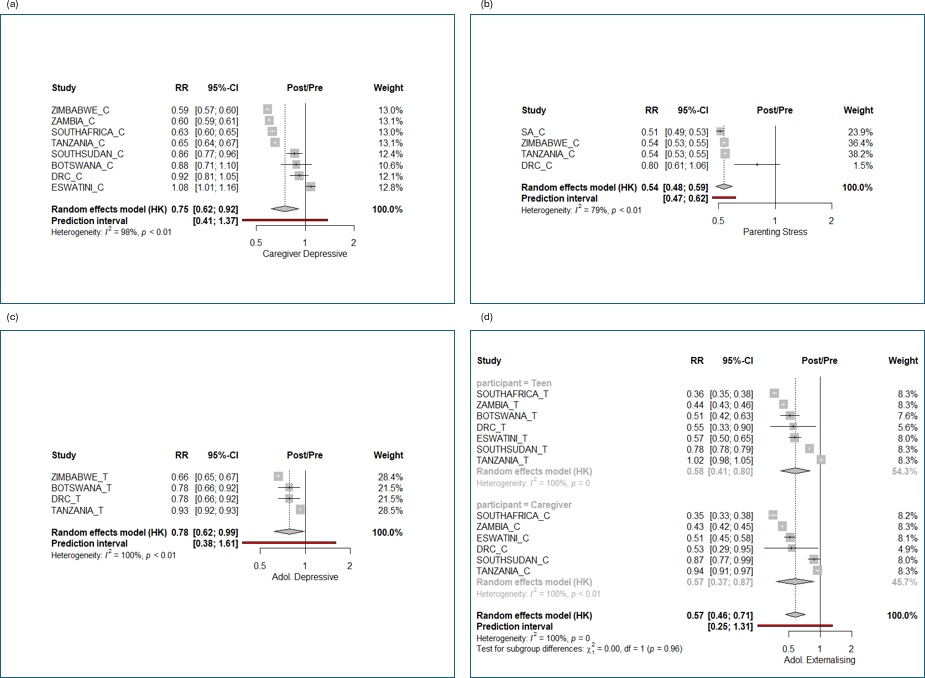
(a) Mental health: meta-analytic estimates of change by country in scores from pre-intervention to post-intervention in caregiver depression. (b) Mental health: meta-analytic estimates of change by country in scores from pre-intervention to post-intervention in parenting stress. (c) Mental health: meta-analytic estimates of change by country in scores from pre-intervention to post-intervention in adolescent depression. (d) Mental health: meta-analytic estimates of change by country in scores from pre-intervention to post-intervention in adolescent externalising behaviour. RR = Risk Ratio, in this analysis measuring the proportional change in the measurement score from pre- to post-intervention.

**Figure 5:**
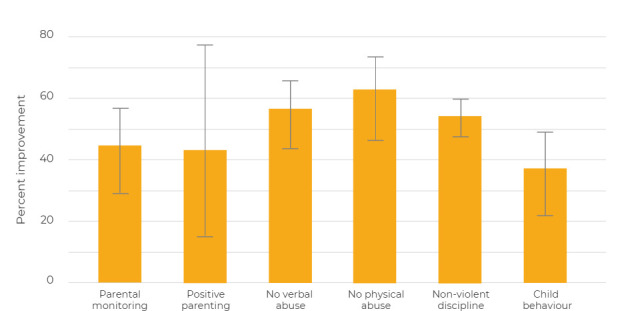
Percent improvement in scores from pre-intervention to post-intervention, using combined ratios across all countries in meta-analyses. To create this visual summary of the impact of the intervention across the different scores, some of the combined effects of post-intervention to pre-intervention ratios as estimated by the meta-analysis were reversed so that all effects could be expressed in terms of “improvement”. The proportional post- to pre- ratios were multiplied by 100 to obtain a percentage improvement. The height of bars indicate the percentage improvement and the connected horizontal lines the 95% confidence intervals for these improvements.

### Violence against children

*Physical abuse*. Overall, the estimated decline in physical abuse scores across countries reflected a decrease of 65% (95% CI 51% to 74%) (figure 2a). Declines in all countries ranged between 32% (in Eswatini adolescent reports) and 87% (in Zimbabwe adolescent reports). Adolescent and caregiver reports validated each other in all countries, in that they indicated the same directional change.

*Emotional abuse*. Overall, the estimated decline in emotional abuse scores was 59% (95% CI 48% to 68%) (figure 2b). Declines in all countries ranged between 25% (in DRC caregiver reports) and 80% (in Zimbabwe adolescent reports). Adolescent and caregiver reports validated each other in all countries.

*Approval of corporal punishment*. Overall, the estimated decline was 55% (95% CI 48% to 60%) (figure 2c). Declines in all countries ranged between 35% (Eswatini) and 68% (South Sudan) among adolescents and between 25% (DRC) and 68% (Tanzania) among caregivers. Adolescent and caregiver reports validated each other in all countries.

We noted that there was substantial heterogeneity in pre-intervention levels of physical and emotional abuse across the eight countries (online supplemental figure 1). For example, adolescent-reported mean physical abuse scores ranged between 0.64 and 5.19 and mean emotional abuse scores ranged between 1.2 and 3.6. Changes in rates of violence also varied across countries; however, despite this heterogeneity, there was an improvement in all countries. In all countries except for Eswatini, caregiver and adolescent responses validated each other, with closely aligned patterns of improvement.

#### Parenting

*Positive parental involvement.* Overall, the estimated improvement across all countries was 39% (95% CI 8% to 80%) (figure 3a). Improvements in all countries ranged between 14% and 2.4-fold (with exceptions of Tanzania and Eswatini caregivers). Adolescent and caregiver reports validated each other in all countries except for Eswatini, where adolescents reported improvements in parenting, but caregivers did not.

*Poor supervision/monitoring*. Overall, the estimated reduction across all countries was 48% (95% CI 34% to 58%) (figure 3b). Declines in all countries ranged between 6% (South Sudan caregivers) and 70% (Zimbabwe adolescents). Adolescent and caregiver reports validated each other in all countries.

We noted that pre-intervention positive parental involvement was consistently low across the different countries (online supplemental table 2a) with a mean score of 1.39 (95% CI 1.08 to 1.8) while pre-intervention supervision scores were more variable, ranging between 0.07 (Botswana adolescents) and 7.55 (DRC caregivers, with higher scores showing more supervision) (online supplemental figure 2b).

#### Mental health

*Caregiver depressive symptoms*. Overall, the estimated reduction across all countries was 25% (95% CI 8% to 48%). However, this outcome showed some heterogeneity (figure 4a): depressed mood scores decreased between 41% and 14% in Zimbabwe, Zambia, South Africa, Tanzania and South Sudan but showed no change in Botswana and the DRC, and an increase of 8% (95% CI 1% to 16%) in Eswatini. *Parenting stress*. Overall, the estimated reduction across all countries was 46% (95% CI 41% to 52%). Parenting stress was only measured in four countries (figure 4b) and showed a just <50% decline in South Africa, Zimbabwe and Tanzania, with a smaller decline of 20% in the DRC. *Adolescent depressive symptoms*. Overall, the estimated reduction across all countries was 22% (95% CI 1% to 38%). Depression symptoms were only measured in four of the countries (figure 4c) and showed a decline of between 33% (Zimbabwe) and 7% (Tanzania). *Adolescent externalising behaviour*. Overall, the estimated reduction across all countries was 43% (95% CI 29% to 54%) (figure 4d). Declines in all countries ranged between 64% and 43%, except for Tanzania, where no change was observed, and to a lesser extent in South Sudan (22% decline).

We note that the two countries with relatively higher pre-intervention assessments of adolescent externalising behaviour showed the smallest declines (online supplemental figure 3d). Pre-intervention levels of caregiver depression showed heterogeneity, ranging between 0.42 in South Sudan and 5.17 in the DRC (online supplemental figure 3a), and pre-intervention levels of parental stress ranged between 2.53 in Tanzania and 4.44 in the DRC (online supplemental figure 3b). Pre-intervention levels of adolescent depression had lower heterogeneity, ranging from 2.87 in Zimbabwe to 3.83 in Botswana and the DRC (online supplemental figure 3c).

## Discussion

This study assessed real-world scale-up of an evidence-based parenting programme across eight Southern, Eastern and Central African countries, within routine services of government, local and international NGO delivery. Multi-country meta-analysis using caregiver and adolescent report of standardised items showed positive improvements across countries on all outcomes: reductions in physical and emotional violence, improvements in positive parenting and parental supervision, reductions in caregiver depressive symptoms and parenting stress and reductions in adolescent depressive symptoms and externalising behaviour.

There was heterogeneity between countries in both pre-intervention scores and extent of change. This was anticipated due to variation in economic and political conditions and socio-cultural values and practices across the region, and to differences in recruitment of participants across a range of organisations. We note that parenting programmes were delivered in a range of challenging conditions, including conflict, COVID-19 and extreme climate events. These factors no doubt affected (at least) parenting stress and mental health, as well as service delivery. Perhaps related to this, findings showed differences across countries in terms of baseline scores of outcomes such as physical and emotional violence, and also differences in the extent of observed change. It is difficult to establish levels of clinical significance in a study that spans multiple countries, cultures, languages and populations. There is also no global agreement on what extent of reduction in childhood violence is a meaningful one, and how that may differ in a population-level programme relative to an indicated programme. However, we note that findings showed remarkable consistency in reported significant, positive effects, with a small number of exceptions, across countries and between caregiver and adolescent reports.

The study has important limitations. First, as countries collected their own data, this gives a tentative overall effect size based on a set of heterogeneous studies with a common intervention and measures, but many other differences as expected across real-world delivery systems. Second, as an implementation study in routine settings with limited resources, this did not include control groups, thus limiting any claims of causality. However, we note that improvements across outcomes were similar to those in randomised trials of evidence-based parenting programmes.^3^ Third, surveys were collected immediately post-intervention, and we would expect attenuation of some effects over time.^25^ Fourth, the data collected did not allow for accounting for clustering by location within countries. Fifth, of the 14 countries invited to join the study, six declined, and possible response bias at this level is unknown. Sixth, sample sizes differed across countries and were lower in humanitarian contexts, but all countries had sufficient samples for analyses.

This study prioritised strengthening local capacity to evaluate interventions using robust measures and ethical research methods. We followed principles of research equity^39^ with local partners by sharing funding and supporting them to clean, analyse and publish with lead authorship (where desired) from their data, which was retained and stored locally.^30 40^ If we are to develop a robust evidence base for scaled-up services, building research skills and resources among implementers is an important step.

We aimed to begin to answer an essential question for policy and programme delivery: whether a widely used evidence-based parenting programme works at scale. Findings show substantial positive changes on violence prevention, parenting and mental health, in both caregiver and adolescent reports. Despite expected heterogeneity in pre-intervention and post-intervention scores across countries, it was possible to identify overall positive impacts across 120 000 participants in eight countries in Africa, and through a pandemic, humanitarian crises and climate hazards. Findings suggest that evidence-based parenting programmes are able to retain effectiveness from randomised trial conditions across the multiple complex and challenging contexts of real-world implementation.

## Supplementary material

10.1136/bmjgh-2025-020422 online supplemental file 1

10.1136/bmjgh-2025-020422 online supplemental file 2

10.1136/bmjgh-2025-020422 online supplemental file 3

## Data Availability

Data are available on reasonable request.
