# Supplementary material for: Are parenting programmes effective at scale? Associations with violence against adolescent girls, parenting and mental health in real-world delivery across eight African countries: a meta-analysis of pre-post surveys
Source: BMJ Glob Health. 2026 May 5;11(5):e020422. doi: 10.1136/bmjgh-2025-020422 (PMC13141112; doi:10.1136/bmjgh-2025-020422)
Supplement: online supplemental file 1 [file bmjgh-11-5-s001.docx]

Supplementary materials:

Figure S1: Violence against children: Meta-analytic estimates by country of Pre-intervention scores in physical abuse (1a) and emotional abuse (1b)

Figure S2: Parenting: Meta-analytic estimatesby country of Pre-intervention scores in positive involved parenting (2a) and poor parental monitoring/supervision (2b)

Figure S3: Mental health: Meta-analytic estimates by country of Pre-intervention scores in caregiver depression (3a), parenting stress (3b), adolescent depression (3c) and adolescent externalising behavior (3d)

Figure S4(a): Bar graphs for ICAST1 measurement items used to calculate Physical and Emotional Abuse scores by country and participant type pre and post the intervention


Figure S4(b): Bar graphs for APQ measurement items used to calculate Supportive Parenting and Poor Superivsion Scores by country and participant type pre and post the intervention

Figure S4(c): Bar graphs for CESD measurement items used to calculate Caregiver Depressive Score and PSS measurement items to calculate Parental Stress Score by country and participant type pre and post the intervention


Figure S4(d): Bar graphs for SDQ measurement items used to calculate Adolescent Externalising Scores and CESD measurement items to calculate Adolescent Depressive Scores by country and participant type pre and post the intervention

Table S1(a) Cross sectional summary statistics for Physical Abuse, Emotional Abuse, Corporal Punishment and Parenting Stress Scores by country and participanttype pre and post intervention

Table S1(b) Cross sectional summary statistics for Positive Parenting, Poor Supervision, Adolescent Externalising and Depressive Scores by country and participanttype pre and post intervention
