# Supplementary material for: Are parenting programmes effective at scale? Associations with violence against adolescent girls, parenting and mental health in real-world delivery across eight African countries: a meta-analysis of pre-post surveys
Source: BMJ Glob Health. 2026 May 5;11(5):e020422. doi: 10.1136/bmjgh-2025-020422 (PMC13141112; doi:10.1136/bmjgh-2025-020422)
Supplement: online supplemental file 2 [file bmjgh-11-5-s002.docx]

**Supplement: Data management**

**Figure 1:** *Overview of data management process undergone before analyses.*

Figure 1 summarises the different processes undergone during the management of the data prior to the analyses. To elaborate on this, the procedure [^5^](#bookmark2) executed on the data collected from each country is detailed below.

**Material collation**

This process involved the collation of all tools (e.g., the pre and post assessments) and other relevant documents (e.g., the context-specific protocols) used for the purpose of intervention implementation and/or evaluation. In some countries, these surveys were adapted via translation (e.g., presented to participants in local language), and so required re-translation into English.

**Label standardization**

The anonymized data received was often in an unprocessed format, where, for example, the entire question posed to participants in the pre-post surveys was written out and regarded as the variable name. For the sake of simplicity, these sentence-based variable names were instead replaced with a more concise description capturing the scale (e.g., ICAST) and the scale item (e.g., spank) to form one short abbreviated form (e.g., ICAST_spank). Thereafter, country-specific codebooks were created to track the specific scales and the associated items surveyed for caregivers as well as adolescents. Across countries, the similarities and differences in the scale items asked in the participants’ assessments were noted.

**Format standardization**

Similar to the variable name, the responses of these were also mostly provided in a format not appropriate for analyses. For instance, the item responses in the data would present as character strings (e.g., very true) instead of as a numeric value (e.g., 2). The alteration in formatting (from character string to number) is necessary for the analyses (e.g., computing the composite variable for the purpose of fitting models). Across all countries, the same universal range of responses for each item within each scale was prevalent, hence no adjustment was required in this respect.

**Range validation**

The range of responses possible for items within each scale were validated. In other words, any impossible values were eliminated by coding the response as missing (NA) instead. For example, if the SDQ-scale items’ responses are expected to be one of three viable values, zero (0 = *not true*), one (1 = *somewhat true*) and two (2 = *very true*), then the presence of an implausible numeric value (outside of the range ∈ {0*,* 1*,* 2} or any non-integer number) was corrected for. These discrepancies, which are most likely the result of a data capturing error, are necessary to rectify in order to avoid any potential biases in subsequent analyses. This process was not only followed for scale item responses, but also demographic information. For example, adolescent who were recorded as being younger than nine and older than eighteen [^6^](#bookmark3) were removed.

**Reverse coding**

Certain items necessitated re-coding such that these would align with the same sentiment or construct direction of its specific Likert scale. For instance, consider the SDQ scale where its items relay information about problem- atic, not praise-worthy/acceptable, behaviours exhibited by adolescents. Hence, the higher the score, the more behavioural issues. Given this general negative sentiment, any items (like SDQ_obedient) which contradict this (i.e., suggestive of less behavioural issues) conceptual direction needs to be changed via the following general formula for each participant: *r_ip_* = (max*_i_* + min*_i_*) - *o_ip_*, where *r_ip_* and *o_ip_* are the reversed and original score of item *i* for participant *p*, respectively, whilst max*_i_* and min*_i_* are the theoretical maximum and minimum possible values of the item *i* within a specific scale. Through this process of inverting the response scale for specific survey items, consistency of interpretation is ensured - this is especially crucial when creating composite variables. It is noted that these reverse-worded items within a scale are typically added in a survey to prevent response biases (like straight-lining[^7^](#bookmark4)). This is also appropriate to account when assessing the reliability (or internal consistency) of a scale (for example, via Cronbach’s Alpha Coefficient).

For both the range validation and reverse coding steps, functions from the psych package, such as describe and reverse.code, were employed respectively.

**Duplication removal**

The presence of duplicate responses (e.g., the responses of the same caregiver/adolescent were doubly captured) were searched for and removed. This was achieved by checking for identical responses across all scales, con- ditioned on the caregiver/adolescents’ identifying number (or alternatively a combination of their demographic information, like age, sex, highest level of education attained, and/or response to risk factors).

**Composite variable creation**

Composite variables, or summed scores of individual items within each separate scale (or subscale) measuring a certain construct, was created for each individual caregiver and adolescent. These summed scores, the response

or dependent variable, formed the basis of the data to be analysed (firstly via mixed-effect models, and then subsequently the results thereof forming part of the meta-analysis). Though, it is mentioned that for the scale measuring attitude towards corporal punishment, no composite variable creation was necessary due to it comprising of only one item.

**Response variation**

The general patterns in the responses were investigated. The focus here was to check for lack of variation to an extreme extent (e.g., the same response was filled out by an adolescent for all items from some or most scales). For this, summary statistics were explored - if a participant presented with no or near-zero variance in response across scale-specific items, the data was manually checked to determine for the presence of the straight-lining phenomenon. Alternatively, this was checked with mode frequencies - one type of response perhaps dominated and was selected for all items irrespective of the scale (e.g., the extreme negative sentiment was consistently chosen, like *never* for all ICAST, APQ, and PSE-scale items as well as *not true* for SDQ-scale items). Patterns of alternating responses (e.g., *never*, *always*, *never*, *always*) were also checked via an autocorrelation calculation or simply counting the number of unique responses. All of the aforementioned, was further corroborated with visualizing heatmaps (desirable to see some differentiation in colour) of the participants’ responses across all scale-items. In the event of there existing an extreme suspicion of straight-lining (or some other extreme form of response uniformity), all data from these affected participants were disregarded.

**Final inspection**

In this penultimate step of data management, random checks were manually performed to further validate the successful execution of the previously outlined procedures. This process was repeated on ten different occasions.

**Matching**

Finally, as part of managing the data, different datasets were derived for different potential analysis’ purposes based on different matching approaches.

Two matching approaches were considered: *Time-matching*[^8^](#bookmark5), where the pre-assessment of a caregiver (adolescent) is matched to their own post-assessment, and *Dyad-matching*, where the caregiver is matched to their corresponding adolescent.

In order to apply both types of matching, a unique identification number (or ID [^9^](#bookmark6)) assigned to each caregiver and each adolescent is relied upon. Though, in some countries (especially for Eswatini and South Sudan) there were non-unique identification numbers present in the data. This in turn meant that, for instance, one caregiver would be linked to two pre-assessments and three post-assessments. Initially, these were suspected to be the result of data- capturing errors and were in fact simply duplicates of one another. However, there were no similarities between these repeated measures, in terms of demographic information and/or item response patterns. Consequently, it was decided to remove all data related to these participants with non-unique IDs.

1. For the Botswana SW-CRT data, this matching of assessments for a particular participant was extended from not only pre and post assessments, but additionally the baseline and endline ones too (where relevant)
2. A variable created as part of the anonymization process before receiving the data.

**Table 1** *Comparison of country-specific caregiver-adolescent dyad numbers before and after the PLH-Teens PIP across the different datasets.*

**Dataset I**^†^ **Dataset II**^†^ **Dataset III**^†^

**Pre Post Pre Post Pre Post**

| **Botswana** |  |  |  |  |  |  |
| --- | --- | --- | --- | --- | --- | --- |
| *Caregiver* | 521 | 438 | 445 | 320 | 223 | 223 |
| *Adolescent* | 530 | 331 | 445 | 232 | 223 | 223 |
| **DRC** |  |  |  |  |  |  |
| *Caregiver* | 166 | 99 | 124 | 44 | 25 | 25 |
| *Adolescent* | 159 | 74 | 124 | 43 | 25 | 25 |
| **Eswatini** |  |  |  |  |  |  |
| *Caregiver* | 1 216 | 1 216 | 603 | 603 | 603 | 603 |
| *Adolescent* | 1 094 | 1 094 | 603 | 603 | 603 | 603 |
| **South Sudan** |  |  |  |  |  |  |
| *Caregiver* | 290 | 290 | 34 | 34 | 34 | 34 |
| *Adolescent* | 295 | 295 | 34 | 34 | 34 | 34 |
| **Tanzania** |  |  |  |  |  |  |
| *Caregiver* | 30 644 | 30 528 | 28 355 | 28 221 | 28 221 | 28 221 |
| *Adolescent* | 28 373 | 28 373 | 28 355 | 28 355 | 28 221 | 28 221 |
| **Zambia** |  |  |  |  |  |  |
| *Caregiver* | 14 040 | 11 607 | 14 040 | 11 607 | 11 607 | 11 607 |
| *Adolescent* | 14 040 | 11 607 | 14 040 | 11 607 | 11 607 | 11 607 |
| **Zimbabwe** |  |  |  |  |  |  |
| *Caregiver* | 9 981 | 9 972 | 6 664 | 6 614 | 6 461 | 6 461 |
| *Adolescent* | 9 801 | 9 789 | 6 664 | 6 547 | 6 461 | 6 461 |

**Note.** **: unmatched;* †*: matched at pre only; ♯: matched at pre and post*

Table 1 outlines the contrasting country-specific sample sizes incurred as a result of applying different matching approaches to the data.

Furthermore, the derived datasets are described as:

- *Dataset I - unmatched*, where data from all caregivers and all adolescents are collated, regardless of dyad- matching and time-matching. This means that caregivers do not necessarily have to be matched to their adolescent (i.e., adolescent data is allowed to be missing, which could be the case due to a data-capturing error), and vice versa. Moreover, the one of the assessments (pre or post) for a caregiver (or adolescent) is also allowed to be missing.
- *Dataset II - matched at pre only*, where caregiver and adolescents data has undergone dyad-matching only.
- *Dataset III - matched at pre and post*, where caregiver and adolescent data has been subjected to both dyad-matching and time-matching.

Comparing Dataset II and Dataset III, it is seen that there is sample size consistency (same number of caregiver- s/adolescents retained for Eswatini and South Sudan). For Zambia, only caregiver data is lost after time-and-dyad matching.

From Dataset I, it is evident that Tanzania (and Zambia, though to a lesser extent in comparison) reached a significantly greater number of caregivers and adolescents. This is likely due to more actively-involved research support teams and less limitation on resources, in terms of funding and number of facilitators, implicated during the implementation of intervention. The lack of the aforementioned, in addition to the countries’ past and current strained social/political/economical context (Usanov et al., 2013; Kiros, 2024), might also explain the limited data collected from the DRC and South Sudan (respectively). The latter might affect the generalizability of the analyses for these two countries.

In general, there is quite a significant amount of data attrition as more stringent forms of matching are applied. This is particularly pronounced for the DRC and South Sudan, where only less than 20% of the original data is retained (in Dataset III). On the other hand, it is shown that for countries (Tanzania and Zambia) with the largest sample sizes data retainment was high (over 80%). This finding might be an artefact of the smoother implementation process (e.g., again as a result of better access to resources) of the intervention in these two latter countries, compared to DRC and South Sudan. Moreover, loss in data is also noted when comparing the sample sizes before and after the intervention, as well as across caregiver and adolescents, both within a country and across the different datasets. As expected, there exists uniformity in the numbers for Dataset III, which provides assurance that the matching approaches were aptly applied.

For the purpose of this study, Dataset I was used for all analyses. Here, it was assumed that all caregivers and all adolescents received the PLH-Teens PIP, and so the need to use dyad-matched datasets was not deemed necessary. Additionally, it was aimed to avoid any potential biases in the analyses which could arise from ex- cluding so-called imperfectly-matched data, especially if this information came from countries with very volatile political and socio-economic realities riddled with civil or external conflict (i.e., participating in a PIP is not the forefront priority within the community). Furthermore, the lack of time-matching was also considered unproblematic given that such pre/post-assessment missingness would be dealt with through model-based imputation via maximum likelihood estimation (under the assumption of missing at random).
