# Supplementary material for: Are parenting programmes effective at scale? Associations with violence against adolescent girls, parenting and mental health in real-world delivery across eight African countries: a meta-analysis of pre-post surveys
Source: BMJ Glob Health. 2026 May 5;11(5):e020422. doi: 10.1136/bmjgh-2025-020422 (PMC13141112; doi:10.1136/bmjgh-2025-020422)
Supplement: online supplemental file 3 [file bmjgh-11-5-s003.docx]

### BMJ Global Health Author Reflexivity Statement

Adapted from Morton, B., Vercueil, A., Masekela, R., Heinz, E., Reimer, L., Saleh, S., Kalinga, C., Seekles, M., Biccard, B., Chakaya, J., Abimbola, S., Obasi, A. and Oriyo, N. (2022), Consensus statement on measures to promote equitable authorship in the publication of research from international partnerships. Anaesthesia, 77: 264-276. <https://doi.org/10.1111/anae.15597>

| **Study conceptualisation** | |
| --- | --- |
| 1. How does this study address local research and policy priorities? | This study responds to priorities identified by African governments, NGOs, and multilateral agencies to understand whether evidence-based parenting programmes remain effective when delivered at scale through routine service systems. Implementing partners across eight countries highlighted the urgent need for locally relevant evidence on violence prevention, parenting, and adolescent wellbeing in real-world settings, including humanitarian and pandemic-affected contexts. The research directly supports regional policy commitments made under the Global Initiative to Support Parents and the African Partnership to End Violence Against Children, and aligns with country-level goals to integrate parenting support into existing services and systems. |
| 1. How were local researchers involved in study design? | Local research partners and implementing organisations in each participating country contributed to ensuring that design and measures were feasible, culturally appropriate, and aligned with ongoing programme delivery. The study used secondary analysis of routinely collected pre-post survey data, which were embedded within partner organisations’ ongoing service delivery. Partners reviewed and adapted survey instruments, led translation and back-translation processes, and advised on ethical procedures and consent materials. Local teams co-led capacity-building workshops to support embedded monitoring and evaluation systems and contributed to the development of the study protocol. |
| **Research management** | |
| 1. How has funding been used to support the local research team(s)? | Funding from multiple international and national sources, including the European Research Council, UKRI GCRF, Research England, MRC UK, the National Research Foundation of South Africa, Oak Foundation, and the LEGO Foundation, supported institutions in both the Global South and North. Implementing partners in each country received funding and training to support data collection activities. All partners retained ownership of their own primary data. |
| **Data acquisition and analysis** | |
| 1. How are research staff who conducted data collection acknowledged? | All pre-post survey data were collected by trained staff from partner organisations as part of the organisation’s routine programme monitoring.  Each organisation’s contribution is recognised in the acknowledgements section of the manuscript. The project lead/s from each organisation are also included as co-authors. |
| 1. How have members of the research partnership been provided with access to study data? | Each partner organisation retained ownership of its own primary data and shared only pseudonymised datasets for pooled analyses through formal data-sharing agreements. All partners have ongoing access to cleaned datasets and analytic outputs relevant to their country. |
| 1. How were data used to develop analytical skills within the partnership? | Country teams participated in capacity-building workshops on data management, cleaning, and analysis using statistical software. Joint analytic sessions supported partners to interpret their findings and integrate these into country-specific reports, strengthening local monitoring and evaluation systems. |
| **Data interpretation** | |
| 1. How have research partners collaborated in interpreting study data? | For this wider project, interpretation was conducted collaboratively through iterative discussions between country partners and academic teams. Country teams contextualised results to their local implementation conditions, and comparative discussions across sites helped explain variations. Draft interpretations were jointly reviewed to ensure that findings reflected both local experiences and cross-country perspectives. |
| **Drafting and revising for intellectual content** | |
| 1. How were research partners supported to develop writing skills? | Writing was coordinated through shared drafts circulated across all partners, including implementing organisations. Partners were encouraged to contribute to the manuscript and so develop their writing skills. |
| 1. How will research products be shared to address local needs? | Findings will be disseminated through open-access publication, country briefs, webinars, and presentations with national stakeholders. Country partners are supported to use the results for programme planning, fundraising, and policy advocacy, ensuring that outputs have local practical value. |
| **Authorship** | |
| 1. How is the leadership, contribution and ownership of this work by LMIC researchers recognised within the authorship? | Of the 56 authors, 49 are based at or affiliated with institutions in low- and middle-income countries across Sub-Saharan Africa, while 7 are based solely in high-income countries. This distribution reflects the study’s design as a secondary analysis of routinely collected data from implementing partners in eight African countries. Leadership was shared between the University of Cape Town and the University of Oxford, reflecting the collaboration’s embedded structure across Global South and North institutions. Because data collection, management, and contextual interpretation were led by LMIC-based organisations, authorship recognises their substantive leadership and intellectual contributions, with collaborative support in analysis and writing from partner institutions in the Global North. |
| 1. How have early career researchers across the partnership been included within the authorship team? | Early career researchers from both African and UK institutions were included as co-authors and contributed to data analysis, coordination, and manuscript drafting, providing them with experience in international collaboration and publication. |
| 1. How has gender balance been addressed within the authorship? | Of the 56 authors, 39 are female and 17 are male. The predominance of female authors reflects the composition of the parenting and social service sectors in the participating countries, where the majority of programme implementers and researchers are women. Authorship decisions were made to ensure equitable recognition of all contributions, consistent with principles of fairness and inclusivity. |
| **Training** | |
| 1. How has the project contributed to training of LMIC researchers? | The wider project directly supported training for LMIC researchers and programme staff in research ethics, data management, statistical analysis, and academic writing. This training was embedded in implementation, enabling skills transfer and long-term capacity building within partner organisations. |
| **Infrastructure** | |
| 1. How has the project contributed to improvements in local infrastructure? | The study contributed to local research infrastructure through strengthening data collection systems and data-sharing protocols. These systems now support ongoing programme evaluation and future research within participating organisations. |
| **Governance** | |
| 1. What safeguarding procedures were used to protect local study participants and researchers? | Data collection was integrated within routine service delivery by established implementing organisations, as part of their ongoing monitoring and evaluation processes. Each organisation had existing safeguarding and referral procedures in place. These organisations maintained their own protocols for responding to disclosures of violence, abuse, or distress. Field staff were trained in ethical data collection, confidentiality, and safe handling of sensitive information. Ethical approval was obtained from the University of Oxford, the University of Cape Town, and national ethics committees in all participating countries, ensuring oversight of both participant and researcher safety. |
